# Vaccine Attitudes, Knowledge, and Confidence Among Nursing, Pediatric Nursing, and Midwifery Undergraduate Students in Italy

**DOI:** 10.3390/vaccines13080813

**Published:** 2025-07-30

**Authors:** Ersilia Buonomo, Daniele Di Giovanni, Gaia Piunno, Stefania Moramarco, Giuliana D’Elpidio, Ercole Vellone, Enkeleda Gjini, Mariachiara Carestia, Cristiana Ferrari, Luca Coppeta

**Affiliations:** 1 Department of Biomedicine and Prevention, University of Rome “Tor Vergata”, 00133 Rome, Italydaniele.di.giovanni@uniroma2.it (D.D.G.); ercole.vellone@uniroma2.it (E.V.); mariachiara.carestia@uniroma2.it (M.C.); cristiana.ferrari@ptvonline.it (C.F.); luca.coppeta@ptvonline.it (L.C.); 2 Faculty of Medicine, Catholic University of “Our Lady of Good Counsel”, 1000 Tirane, Albania; enkeleda.shkurti@umed.edu.al; 3 Pediatric Nursing School, Bambino Gesù Children Hospital IRCCS, 00163 Rome, Italy; giuliana.delpidio@opbg.net; 4 Faculty of Nursing and Midwifery, Wroclaw Medical University, 51-618 Wrocław, Poland; 5 PhD Program in Social, Occupational and Medico-Legal Sciences, Department of Occupational Medicine, University of Rome Tor Vergata, Viale Oxford 81, 00133 Rome, Italy

**Keywords:** vaccine hesitancy, vaccine confidence, nursing students, midwifery, pediatric nursing, vaccine literacy, COVID-19 vaccination, health education, healthcare students

## Abstract

Background: Vaccine hesitancy (VH) represents a growing concern among healthcare professionals and students, potentially undermining public health efforts. Nursing, pediatric nursing, and midwifery students are future vaccinators and educators, making it essential to understand their attitudes, knowledge, and confidence toward vaccination. This study aims to assess vaccine-related perceptions and behaviors among these student populations in an Italian university. Methods: A cross-sectional survey was conducted between November 2022 and February 2024 at the University of Rome “Tor Vergata”. A structured, anonymous questionnaire, including the Vaccination Attitudes Examination (VAX) scale, vaccine knowledge items, and sources of information, was administered to students in nursing (*n* = 205), pediatric nursing (*n* = 46), and midwifery (*n* = 21). Statistical analyses included descriptive statistics, ANOVA, post hoc tests, and Mann–Whitney U tests. Results: Among the 272 participants, 20.6% reported refusing at least one recommended vaccine, and 18.4% delayed vaccination for non-medical reasons. Vaccine knowledge and confidence increased significantly with academic progression (*p* < 0.001). Midwifery students showed both the highest concern for long-term vaccine effects and the greatest confidence in vaccine safety. Institutional and scientific sources were the most trusted, though traditional and non-institutional media also influenced perceptions, particularly among midwifery students. Conclusions: Despite high COVID-19 vaccine uptake, VH persists among health professional students. Discipline-specific patterns highlight the need for early, targeted educational strategies to enhance vaccine literacy and reduce hesitancy. Tailored training may empower future professionals to become informed and credible advocates for vaccination.

## 1. Introduction

Vaccination represents one of the most powerful and cost-effective tools in public health, contributing to the prevention of an estimated 3.5 to 5 million deaths each year worldwide [1]. By reducing the burden of vaccine-preventable diseases, immunization not only protects individuals but also promotes herd immunity, reducing transmission in communities and safeguarding vulnerable populations [2]. The success of vaccination campaigns depends not only on the availability of vaccines but also on widespread acceptance, particularly among healthcare workers and those who are being trained to become future healthcare professionals, who serve as both role models and key communicators in the healthcare system [3,4,5].

University students enrolled in health-related programs are critical to the future success of vaccination strategies. As the next generation of healthcare providers, they will be responsible for vaccine administration, patient education, and infection prevention. Their vaccine confidence and knowledge will directly influence public perceptions and coverage rates [6]. Even before that, healthcare degree programs—such as nursing, pediatric nursing, and midwifery—comprise both academic and clinical components. Through clinical placements, students engage directly with patients and at-risk populations. Consequently, they face many of the same vulnerabilities as healthcare professionals, underscoring that the challenges of managing the healthcare workforce also encompass undergraduate students [7]. Therefore, investigating vaccine-related attitudes during their academic training is essential.

While vaccine hesitancy (VH), defined by the World Health Organization (WHO) as the “delay in acceptance or refusal of vaccination despite the availability of vaccination services” [8], has been widely documented among healthcare workers (HCWs) [9,10], and growing evidence shows that it is also present among healthcare students, along with low vaccine confidence (VC) [11]. Given that the success of vaccination efforts also depends on how HCWs are trained about vaccines and vaccinations [12], addressing VH at this early stage is crucial, as it allows for targeted educational interventions aimed at shaping informed, confident professionals. One promising strategy to address VH is the promotion of vaccine literacy, defined as the ability to access, understand, and critically evaluate vaccine-related information in order to make informed decisions [13,14]. Vaccine literacy is especially important in the early stages of professional training, as it influences not only personal health behaviors but also future clinical practice. Given that attitudes toward vaccines can evolve throughout the course of study [15], monitoring hesitancy and its determinants during university training is a necessary step toward building trust and resilience against misinformation [16].

When considering different health sectors, nurses and midwives are often more hesitant about vaccines than physicians, likely due to differences in training and knowledge about vaccines [17,18]. Research has shown that nurses and midwives generally have lower vaccine knowledge and are more susceptible to misconceptions, particularly regarding vaccine effectiveness and potential side effects [19,20]. Misunderstandings or insufficient knowledge about infections are also often cited as barriers to vaccination [21]. Given their frequent and direct interactions with patients, the attitudes of nurses and midwives can significantly influence vaccine uptake, especially among vulnerable populations. Students in these disciplines, who are in training to assume these roles, also play a critical part in shaping future public health efforts. Similar challenges have been reported in Italy, where increased vaccine hesitancy has been observed among healthcare workers and students [22], highlighting the need for more effective vaccine education and communication strategies early in healthcare training. Understanding the reasons behind vaccine hesitancy among healthcare students is, therefore, critical, as they will play an important role in future healthcare initiatives [23].

The COVID-19 crisis provided a clear example of how vaccination has become central to global containment efforts. The WHO has set a target to vaccinate at least 70% of the global population, focusing particularly on healthcare workers and vulnerable groups [24]. However, a growing number of people remain hesitant to be vaccinated, influenced largely by media and digital content, which has amplified skepticism [25]. A recent study conducted in an Italian university found that, between March and June 2021, 71% to 78% of students were willing to accept the COVID-19 vaccine [26]. These numbers were lower than the 86% to 92% reported among Italian college students in early 2020 [27] and January 2021 [28]. These changes may reflect shifting public discourse, evolving perceptions of vaccine safety, or methodological differences in assessing VH. Factors such as health literacy, academic year, and personal exposure to COVID-19 have been shown to influence students’ attitudes toward vaccination [26]. In the specific context of the post-pandemic phenomenon of COVID-19 vaccination, nursing and midwifery students’ decision regret regarding the COVID-19 vaccine has been noted [21]. However, specific data on vaccine hesitancy in these student populations remain limited, especially within the Italian context. Vaccine hesitancy among nursing and midwifery students has already been investigated, or is being explored, using tools such as the Vaccine Confidence Index (VCI) [29]. Despite its limitations, including its narrow scope and lack of formal validation, the VCI has proven useful in identifying trends and areas of concern within this population. Nevertheless, further research is needed to better understand the determinants of vaccine hesitancy in these student groups and to develop more robust and contextually appropriate assessment methods.

The aim of this study is to investigate vaccine attitudes, knowledge, and confidence among nursing, pediatric nursing, and midwifery students enrolled at an Italian medical University.

## 2. Materials and Methods

### 2.1. Study Design and Sample

Between November 2023 and February 2024, we conducted an observational cross-sectional study, collecting data through a structured survey aimed at students enrolled in nursing, pediatric nursing, and midwifery programs at the public University of Rome “Tor Vergata”. Participation in the study was entirely voluntary, and the only inclusion criterion was enrollment in one of the aforementioned degree programs. The participants were invited to complete an anonymous, self-administered online questionnaire distributed via the Google Forms platform. Written informed consent was obtained from all participants prior to completing the survey. The first page of the questionnaire included an explanation of the study’s purpose and the objective of the study, the confidentiality of the information collected, and the participants’ right to withdraw from the study at any time. No incentives or compensation were offered for participation. To begin the survey, participants had to confirm that they had read and accepted the consent page. The study received ethical approval from the Ethics Committee of the University of Rome “Tor Vergata” (Rome) (n.210/22, 2022). The present study was conducted as part of the EuCoRVaC “European Alliance for Collaborative Research on Vaccine Confidence in Public and Occupational Health”.

### 2.2. Questionnaire

Following a comprehensive review of the existing literature and previous research, we developed a semi-structured questionnaire organized into several thematic sections.

The initial section was dedicated to collecting socio-demographic information, vaccination history, and health-related factors associated with immunization. Participants were asked about their personal experiences with routine mandatory vaccines (diphtheriae, poliomyelitis, tetanus, hepatitis B virus, measles, pertussis, rubeola, haemophilus influenzae type b, mumps, and chickenpox), previous encounters with COVID-19, and behaviors associated with vaccine uptake.

In the second section, we employed the validated Italian adaptation of the Vaccination Attitudes Examination (VAX) scale [30], which was customized to align with the specific goals of our study. The VAX scale is designed to evaluate vaccine attitudes and levels of vaccine hesitancy among healthcare-related populations. Lower scores reflect more favorable views toward vaccination, while higher scores are indicative of greater hesitancy. This tool captures general perceptions regarding vaccines across four key dimensions: skepticism toward vaccine efficacy, fear of unforeseen long-term effects, distrust in pharmaceutical industry motives, and a preference for natural immunity over vaccination. Additionally, we included a supplementary item assessing participants’ perspectives on their future responsibilities as healthcare professionals: “I consider it my duty as a health professional to educate patients about vaccinations”. The 12 items in this section were rated using a 5-point Likert scale ranging from 1 (“Totally disagree”) to 5 (“Totally agree”). For accurate scoring, four negatively phrased items were reverse-coded during data analysis.

The third section explored the perceived influence of various information sources on participants’ knowledge of infectious diseases and vaccines. Participants were asked to indicate the degree to which different sources, such as institutional and non-institutional websites, discussions with colleagues, conferences, TV, and radio, had influenced their understanding. This section was inspired by prior research on vaccine hesitancy among nursing staff [31]. Responses were collected using a 5-point Likert scale, where 1 indicated “Not at all” and 5 indicated “Very much”.

The fourth section implemented a version of the Zingg and Siegrist scale [32], adapted to suit the study’s context. This validated instrument comprised 10 items with answer options “yes”, “no”, or “I do not know”, aimed at assessing participants’ vaccine-related knowledge. To quantify overall vaccine knowledge, a scoring system was applied in which correct responses received 1 point, while incorrect or “I do not know” answers were assigned 0 points.

### 2.3. Sample Size

Considering the different degree courses, the survey conducted for this study reached the following sample sizes: *n* = 21 students from the midwifery degree program, representing 52.5% of the total enrolled in that course; *n* = 46 students from the pediatric nursing program, accounting for 51.1% of its student population; and *n* = 205 students from the nursing program, representing 47.8% of enrolled students.

For the nursing program, the required sample size was checked assuming a margin of error of ±5 percentage points, a 95% confidence level, and a standard deviation of 0.5, providing a conservative estimate of variance. Based on these parameters, a minimum of *n* = 203 participants was needed for the analysis.

### 2.4. Statistical Analysis

Socio-demographic data and health status information derived from the initial section of the semi-structured questionnaire were reported as a descriptive analysis (numbers and percentages), as a total, and then split by degree course. The results of continuous variables were presented as mean values ± standard deviations (SDs).

When assessing vaccination hesitancy through the VAX scale, Cronbach’s alpha was employed to determine the internal consistency reliability of the measurement items. The overall internal consistency ranged from 0.80 to 0.90, suggesting each item contributed to the overall reliability of the scale [33]. ANOVA tests and post hoc Bonferroni analysis were used to compare differences in mean values of VAX scores between degree courses.

Differences in vaccine knowledge were observed between students in the early-year (first and second year) group and those in the later-year (third year and beyond) group using the Mann–Whitney U test.

IBM SPSS Statistics (version 27) was utilized for the statistical elaboration of the data.

## 3. Results

A total of *n* = 272 questionnaires were filled in. All invited participants completed the survey, with no withdrawals or refusals registered, resulting in a 100% response rate. Table 1 illustrates the principal socio-demographic characteristics of the study group.

Among the participants, *n* = 242 (89%) were females. Regarding course enrollment, 205 students were from the nursing program (75.4%), *n* = 46 from pediatric nursing (17%), and 21 from midwifery (7.7%). The majority fell within the <25 age range. Specifically, in the nursing course, 56.6% of students were in this age group, while in pediatric nursing and midwifery, the percentages were 63% and 76.2%, respectively.

Concerning religion, *n* = 198 students declared practicing a religious faith (73.9%), with 148 (72.2%) of nursing students, *n* = 37 (80.4%) of pediatric nursing students, and 13 (61.9%) of midwifery students identifying as Catholic Christians. The participants’ nationality was predominantly Italian.

Regarding academic year distribution, in the nursing program, *n* = 145 students (72.3%) were in the first and second years combined, while *n* = 59 students (29.3%) were in the third year or higher (i.e., the beginning of the master’s program). In pediatric nursing, *n* = 46 students (73.9%) were in the first and second years combined, while *n* = 24 students (26.1%) were in the third year. All midwifery students (*n* = 21 students, 100%) were in the third year.

Table 2 presents the vaccination status and vaccine-related health behaviors. In analyzing responses to the health-related behaviors and experiences regarding COVID-19, a high level of engagement with COVID-19-related measures was observed across all groups. Nearly all students (*n* = 271, 99.6%) reported having completed at least one COVID-19 swab test, reflecting widespread testing participation. When asked about contracting COVID-19, 76.5% (*n* = 208) of the total sample had contracted the virus at least once, with midwifery students reporting the highest rate (*n* = 18, 85.7%). Additionally, a significant portion of students (*n* = 180, 66.2%) had taken care of a person with COVID-19, with nursing students showing the highest rate (*n* = 149, 72.7%), indicating their greater exposure to caregiving in the pandemic context.

Regarding vaccination behaviors, almost all students (*n* = 271, 99.6%) had received the COVID-19 vaccine, showing strong adherence to vaccination recommendations. Notably, the percentage of students who had postponed other vaccinations due to the COVID-19 vaccine was relatively low (*n* = 35, 12.9% overall), with slight variations between groups. Midwifery students reported the highest percentage (*n* = 3, 14.3%), which may indicate a more cautious approach to vaccination schedules among them.

The uptake of the HepB vaccine booster was also high, with 78.3% (*n* = 213) of students overall receiving the booster dose. However, there was a noticeable difference between groups, with pediatric nursing students showing the highest uptake at 91.3% (*n* = 42). In contrast, the flu vaccine uptake was considerably lower (*n* = 84, 30.9% overall), with pediatric nursing students showing the highest rate (*n* = 24, 52.2%) and midwifery students showing the lowest (*n* = 3, 14.3%), suggesting varying levels of flu vaccine adherence based on the student group.

In terms of vaccine hesitancy, 20.6% (*n* = 56) of the total sample reported having refused a recommended vaccine at least once, with midwifery students exhibiting the highest refusal rate (*n* = 7, 33.3%). Furthermore, 18.4% (*n* = 50) of students overall reported delaying a vaccination for reasons other than allergy or illness, with pediatric nursing students showing the highest rate of delayed vaccinations (*n* = 14, 30.4%).

Lastly, 11.7% (*n* = 32) of students reported suffering from chronic diseases, with midwifery students reporting the highest rate (*n* = 5, 23.8%), suggesting a potential vulnerability to health complications that might influence vaccine-related behaviors.

Regarding the adapted VAX scale for assessing vaccine hesitancy (VH) among healthcare students, Table 3 provides key insights into responses from different student groups. The “Mistrust of Vaccine Benefit” items were all reverse-scored. A significant difference emerged among the three student groups (*p* = 0.019), with midwifery students reporting the lowest score for the statement “I feel safe after being vaccinated” (1.62 ± 0.60), indicating greater vaccine confidence. Post hoc analysis showed that nursing students had a significantly higher mean score compared to midwifery students (*p* = 0.015). For the item “I can rely on vaccines to stop serious infectious diseases”, the difference among groups was also statistically significant, with pediatric nursing students scoring higher than nursing students (post hoc test *p* = 0.038), suggesting comparatively lower confidence in vaccines’ ability to prevent infectious diseases.

When exploring concerns about unforeseen side effects, a significant variation was found (*p* = 0.011). Midwifery students expressed the highest level of agreement with the statement, “Although most vaccines appear to be safe, there may be problems that we have not yet discovered” (4.43 ± 0.60). The post hoc comparison confirmed that midwifery students were significantly more concerned than nursing students (*p* = 0.008), indicating heightened worry about potential long-term effects. As for perceptions of commercial motives, pediatric nursing students were the least suspicious, giving the lowest score (1.78 ± 0.89) to the item “Vaccines make a lot of money for pharmaceutical companies, but do not do much for regular people”. Meanwhile, nursing students scored significantly higher than midwifery students (*p* = 0.015), implying greater skepticism toward vaccine efficacy. In terms of preference for natural immunity, pediatric nursing students most strongly agreed with the statement, “Natural immunity lasts longer than a vaccination” (2.78 ± 1.21), reflecting a tendency to favor natural over vaccine-induced protection. Finally, when asked about their professional responsibility to educate patients about vaccines, nursing students showed the highest level of agreement (1.47 ± 0.77), highlighting a strong commitment to patient education.

**Figure 1:**
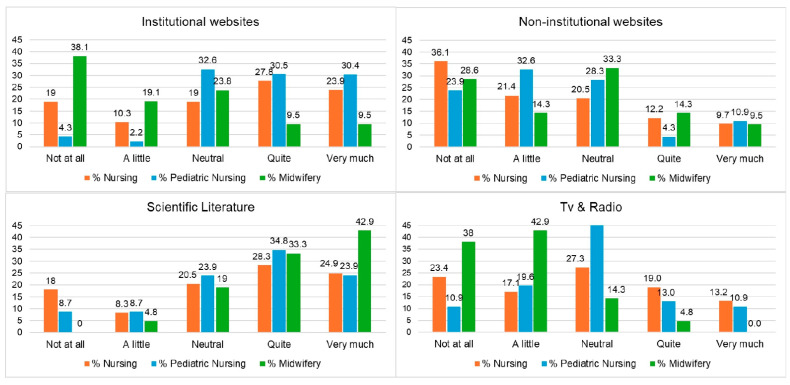
Main sources of information among the sample, divided by degree course.

Figure 1 shows the perceived influence of different information sources by students from nursing, pediatric nursing, and midwifery. Scientific literature was considered “very much” influential by 42.9% of midwifery students (*n* = 9), 24.9% of nursing students (*n* = 51), and 23.9% of pediatric nursing students (*n* = 11), making it the most impactful source overall. TV and radio were rated as “very much” influential by 13.2% of nursing (*n* = 27) and 10.9% of pediatric nursing students (*n* = 5), indicating a still notable role for traditional media. Institutional websites received varied responses: 38.1% of midwifery students (*n* = 8) rated them as “not at all” influential, while 27.8% of nursing students (*n* = 57) and 30.5% of pediatric nursing students (*n* = 14) considered them “quite” influential. There was general agreement among the three student groups that non-institutional websites were not very influential, as a considerable proportion in each group rated them as “not at all” influential, e.g., 36.1% of nursing students (*n* = 74), 28.6% of midwifery students (*n* = 6), and 23.9% of pediatric nursing students (*n* = 11). In summary, scientific sources were the most trusted across groups, while midwifery students appeared more receptive to both traditional media and non-institutional sources.

**Figure 2:**
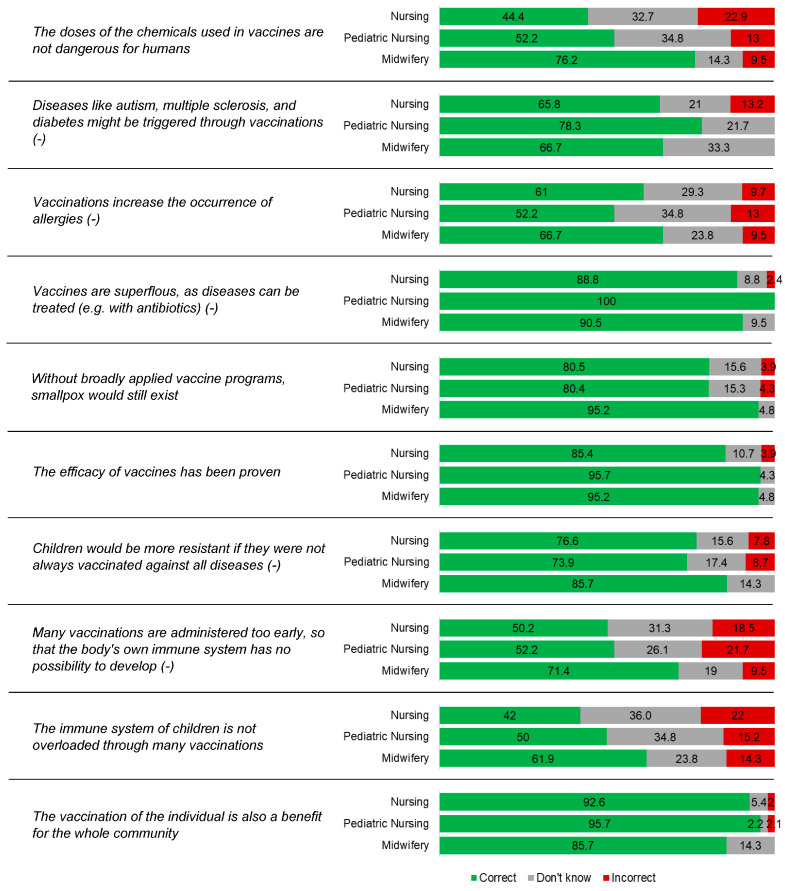
Percentage of correct answers out of the total (*n* = 272), divided by degree course. Items including incorrect statements are marked with (−).

Figure 2 shows questions assessing vaccine knowledge among the students. Overall, midwifery students showed a higher level of awareness regarding the safety of vaccine components, with 76.2% (*n* = 16) correctly stating that excipients in vaccines are not dangerous to humans, compared to 52.2% (*n* = 24) of pediatric nursing and 44.4% (*n* = 91) of nursing students. When addressing the false association between vaccines and autism, 78.3% (*n* = 36) of pediatric nursing students answered correctly, slightly higher than 66.7% (*n* = 14) of midwifery and 65.9% (*n* = 135) of nursing students. Regarding the misconception that vaccines increase the occurrence of allergies, 66.7% (*n* = 14) of midwifery students responded accurately, as did 61.0% (*n* = 125) of nursing and 52.2% (*n* = 24) of pediatric nursing students. Strong agreement on the utility of vaccines was evident, with 100% (*n* = 46) of pediatric nursing students recognizing that vaccines are not superfluous, followed by 90.5% (*n* = 19) of midwifery and 88.8% (*n* = 182) of nursing students. Confidence in vaccine efficacy was also high across all groups, especially among pediatric nursing (95.7%, *n* = 44) and midwifery students (95.2%, *n* = 20), and slightly lower among nursing students (85.4%, *n* = 175). Interestingly, the belief that vaccines are administered too early was rejected more firmly by midwifery students (71.4%, *n* = 15) than by pediatric nursing (52.2%, *n* = 24) and nursing students (50.2%, *n* = 103). Finally, an understanding of community-level benefits of vaccination was widely acknowledged, with 95.7% (*n* = 44) of pediatric nursing, 92.7% (*n* = 190) of nursing, and 85.7% (*n* = 18) of midwifery students agreeing that individual vaccination contributes to the protection of the entire population.

A statistically significant difference in knowledge mean ranks was observed between early-year (first and second year) and later-year (third year) students across all three courses (mean rank: 106.26 for early-year students, *n* = 101; 154.34 for later-year students, *n* = 171; *p* < 0.001). This difference remained statistically significant even when considering only nursing students, indicating a consistent increase in vaccination knowledge with academic progression within the same field of study.

## 4. Discussion

This study investigated vaccine hesitancy (VH), knowledge, and information sources among students in nursing, pediatric nursing, and midwifery programs at an Italian university, offering a focused perspective on discipline-specific attitudes. Overall, the students considered vaccines to be safe, as reflected by the high mean score for the statement “I feel safe after being vaccinated,” indicating strong confidence in vaccines. Additionally, more than 90% of the students responded positively to the statement that the efficacy of vaccines has been proven, a finding also highlighted by Baldolli et al. among healthcare students in France [34].

Although COVID-19 vaccine uptake was very high (99.6%), hesitancy was still present: 20.6% of students reported having refused a recommended vaccine at least once, and 18.4% delayed vaccination for non-medical reasons. Midwifery students showed the highest rate of vaccine refusal (33.3%), consistent with both national and international studies indicating that nurses and midwives often display higher levels of VH compared to medical students or physicians. This tendency is commonly attributed to differences in educational exposure, professional identity, and access to scientific information [16,17,18,20]. These findings resonate with a previous study conducted among Italian and Albanian medical science students [35], in which both groups reported a relatively high degree of trust in vaccines and public health institutions yet continued to express concerns about unforeseen long-term effects. In the present study, such concerns were particularly evident among midwifery students, who, despite expressing the lowest mistrust in vaccine benefits, reported the greatest concern about future adverse effects. This nuanced attitude echoes the concept of “post-vaccine regret” discussed by Tayhan et al. [36].

Regarding vaccine knowledge, our study supports the notion that it improves with academic progression. Indeed, several findings should be interpreted in light of the students’ year of study: those in the later stages of their programs demonstrated greater understanding and acceptance. This is consistent with the results of Berg et al. [37], Barello et al. [27], and Baccolini et al. [26] and aligns with findings from both Albanian and Italian senior medical students, who exhibited higher levels of confidence and knowledge [35]. Similarly, Tavolacci et al. found that students in the early years of their academic careers were more likely to be hesitant [38]. Strengthening vaccine-related knowledge in healthcare students represents a crucial strategy to mitigate vaccine hesitancy among the next generation of healthcare professionals.

Sources of information also played a critical role. While scientific literature and traditional media were generally trusted, some students, particularly in our sample, still reported being influenced by non-institutional websites, reinforcing the concern about misinformation spread, as also emphasized by McCready et al. [23] and previously noted in the cross-national study [35]. In fact, previous Italian studies on healthcare students have shown that those who expressed support for COVID-19 vaccines were more likely to consult institutional websites [39]. Conversely, students who relied primarily on social media as their main source of information were more likely to express greater doubts regarding vaccine effectiveness [40].

Finally, the cross-sectional design of the study provides a timely snapshot of students’ attitudes during their academic training. While it does not permit longitudinal tracking, the observed associations between academic year and vaccine knowledge point to promising avenues for future follow-up research.

### Limitations

While this study provides valuable insights into vaccine attitudes among healthcare students, a few methodological points should be noted. The sample size varied across degree programs, with a higher proportion of nursing students. This reflects enrolment patterns at the institution and enables robust subgroup analysis of the largest cohort. However, it may limit more general conclusions for smaller groups, such as midwifery students.

Another potential limitation of our study is that participation in the survey was voluntary, which may have introduced sample selection bias and limited the generalizability of the findings. It is possible that individuals who chose to participate were those with fewer hesitations. Additionally, since the questionnaires were administered in classrooms to students attending lectures, the coverage rate observed in some courses might be explained by students being engaged in practical training sessions at the time of the survey.

Using a self-administered questionnaire ensured anonymity and encouraged honest responses, although this method is subject to individual interpretation and recall bias. Nevertheless, the structured design and use of validated tools helped to enhance response consistency.

We acknowledge that a limitation of this study is that we did not collect socio-economic factors. Given that it is a public university, we can assume there is a certain degree of variability, especially in vaccine confidence, as highlighted in previous studies [11]. Further investigations, including this information, might be helpful to better understand the specific social, economic, and cultural factors influencing these results.

## 5. Conclusions

Our study confirms that students in health science programs generally perceive vaccines as effective and safe, although some differences were observed across different training courses. These findings underscore the importance of strengthening structured, discipline-specific vaccine education throughout the academic curriculum, starting from the early years of training. Introducing immunization-related content as early as the first year of nursing and midwifery programs is essential to prevent mistrust and misconceptions, which are often fueled by unofficial or unreliable sources of information.

Our study calls for tailored educational strategies, complemented by the development of effective communication skills, both of which are likely to play a crucial role in fostering vaccine confidence among future healthcare professionals. Providing students with accurate, evidence-based knowledge will empower them to act as informed, confident, and credible advocates for vaccination.

Despite these insights, further research is needed to address the persistent gaps in the literature regarding the effectiveness of vaccine education and its long-term impact on healthcare students’ attitudes and behaviors.

## Figures and Tables

**Table 1:** Sample’s socio-demographic characteristics.

	Total (*n* = 272)	%	Nursing (*n* = 205)	%	Pediatric Nursing (*n* = 46)	%	Midwifery (*n* = 21)	%
Female (n.%)	242	(89)	178	(86.8)	43	(93.5)	21	(100)
Age range < 25 years	161	(59.2)	116	(56.6)	29	(63)	16	(76.2)
Academic year (1st and 2nd)	101	(37.1)	67	(32.7)	34	(73.9)	0	(0)
Academic year (>2nd)	171	(62.9)	138	(67.3)	12	(26.1)	21	(100)
Christian religion	201	(73.9)	149	(72.7)	39	(84.8)	13	(61.9)

**Table 2:** Description of the vaccinal status and vaccine-related health conditions of the sample, divided by degree course.

		Total (*n* = 272)	%	Nursing (*n* = 205)	%	Pediatric Nursing (*n* = 46)	%	Midwifery (*n* = 21)	%
I did at least one COVID-19 swab	Yes	271	99.6	204	99.5	46	100	21	100
	No	1	0.4	1	0.5	0	0	0	0
I have contracted COVID-19 disease at least once	Yes	208	76.5	153	74.6	37	80.4	18	85.7
	No	64	23.5	52	25.4	9	19.6	3	14.3
I took care of a person with COVID-19	Yes	180	66.2	149	72.7	20	43.5	11	52.4
	No	92	33.8	56	27.3	26	56.5	10	47.6
A family member of mine contracted COVID-19 disease	Yes	155	93.8	190	92.7	45	97.8	20	95.2
	No	17	6.3	15	7.3	1	2.2	1	4.8
I had the COVID-19 vaccine	Yes	271	99.6	205	100	45	97.8	21	100
	No	1	0.4	0	0	1	2.2	0	0
I postponed at least one other vaccination because of the COVID-19 vaccination	Yes	35	12.9	26	12.7	6	13	3	14.3
	No	237	87.1	179	87.3	40	87	18	85.7
I had my HepB vaccine Booster	Yes	213	78.3	155	75.6	42	91.3	16	76.2
	No	59	21.7	50	24.4	4	8.7	5	23.8
I had the flu vaccine last year	Yes	84	30.9	57	27.8	24	52.2	3	14.3
	No	188	69.1	148	72.2	22	47.8	18	85.7
I have refused a recommended vaccine at least once	Yes	56	20.6	44	21.5	5	10.9	7	33.3
	No	216	79.4	161	78.5	41	89.1	14	66.7
I have delayed a vaccination at least once for reasons other than allergy or illness	Yes	50	18.4	33	16.1	14	30.4	3	14.3
	No	222	81.6	172	83.9	32	69.6	18	85.7
I suffer from chronic diseases	Yes	32	11.7	25	12.2	2	4.3	5	23.8
	No	240	88.3	180	87.8	44	95.7	16	76.2

**Table 3:** Descriptive statistics of the vaccination attitudes, opinions, and confidence about vaccines of the sample. (R) denotes reversed items.

Factor	Item	Total Mean ± SD	Nursing Mean ± SD	Pediatric Nursing Mean ± SD	Midwifery Mean ± SD	*p* Value
Mistrust of vaccine benefit	I feel safe after being vaccinated (R)	2.20 ± 0.98	2.25 ± 1.02	2.22 ± 0.89	1.62 ± 0.60	0.019
	I can rely on vaccines to stop serious infectious diseases (R)	2.48 ± 1.40	2.39 ± 1.43	2.85 ± 1.15	2.57 ± 0.93	0.041
	I feel protected after getting vaccinated (R)	1.71 ± 0.89	1.77 ± 0.95	1.57 ± 0.70	1.43 ± 0.60	0.127
Worries about unforeseen future effects	Although most vaccines appear to be safe, there may be problems that we have not yet discovered	3.81 ± 1.00	3.75 ± 1.05	3.83 ± 0.82	4.43 ± 0.60	0.011
	Vaccines can cause unforeseen problems in children	2.76 ± 1.13	2.80 ± 1.19	2.78 ± 0.94	2.29 ± 0.84	0.139
	I worry about the unknown effects of vaccines in the future	2.92 ± 1.26	2.99 ± 1.30	2.76 ± 1.16	2.57 ± 0.98	0.236
Concerns about commercial profiteering	Vaccines make a lot of money for pharmaceutical companies, but do not do much for regular people	2.06 ± 1.03	2.18 ± 1.06	1.78 ± 0.89	1.52 ± 0.75	0.003
	Authorities promote vaccination for financial gain, not for people’s health	1.94 ± 1.01	2.01 ± 1.03	1.80 ± 0.96	1.52 ± 0.81	0.062
	Vaccination programs are a big conspiracy	1.60 ± 0.92	1.63 ± 0.95	1.48 ± 0.81	1.57 ± 0.87	0.601
Preference for natural immunity	Natural immunity lasts longer than a vaccination	2.59 ± 1.15	2.58 ± 1.12	2.78 ± 1.21	2.29 ± 1.31	0.250
	Natural exposure to viruses and germs gives the safest protection	2.31 ± 1.12	2.37 ± 1.10	2.11 ± 1.12	2.20 ± 1.12	0.305
	I consider it my duty as a health professional to educate patients about vaccinations (R)	1.46 ± 0.78	1.47 ± 0.77	1.41 ± 0.75	1.48 ± 0.98	0.907

## Data Availability

Data are available on request.
